# The Effects of Bradykinin B1 Receptor Antagonism on the Myocardial and Vascular Consequences of Hypertension in SHR Rats

**DOI:** 10.3389/fphys.2019.00624

**Published:** 2019-05-21

**Authors:** Laszlo Deres, Krisztian Eros, Orsolya Horvath, Noemi Bencze, Csongor Cseko, Sandor Farkas, Tamas Habon, Kalman Toth, Robert Halmosi

**Affiliations:** ^1^Medical School, University of Pécs, Pécs, Hungary; ^2^Szentagothai Research Centre, University of Pécs, Pécs, Hungary; ^3^Gedeon Richter Plc., Budapest, Hungary; ^4^1st Department of Medicine, Clinical Centre, University of Pécs, Pécs, Hungary

**Keywords:** hypertensive target organ damages, bradykinin B1 receptor antagonism, NSAIDs, cardiovascular remodeling, echocardiography, spontaneously hypertensive rats

## Abstract

It is known that non-steroidal anti-inflammatory drugs increase cardiovascular (CV) morbidity and mortality. In this study, we examined whether a novel anti-inflammatory drug, bradykinin B1 receptor antagonist (FGY-1153) treatment could influence the development of hypertensive organ damages in spontaneously hypertensive rats (SHR). SHRs were treated with low (FGY-120) or high dose FGY-1153 (FGY-400) and with placebo (Control) for 26 weeks. Wistar–Kyoto rats were used as aged-matched, normotensive controls (WKY). Body weight, food consumption and blood pressure were measured regularly. Echocardiography was performed at the beginning and at the end of the study. Light and electron microscopic analysis of heart and great vessels were performed, and the extent of fibrotic areas was measured. The phosphorylation state of prosurvival Akt-1/glycogen synthase kinase (GSK)-3β pathway and the activation of signaling factors playing part in the fibrotic processes – mitogen activated protein kinases (MAPKs), and TGF-β/Smad2 – were monitored using Western-blot. Body weight and food consumption as well as the elevated blood pressure in SHRs was not influenced by FGY-1153 treatment. However, both doses of FGY-1153 treatment decreased left ventricular (LV) hypertrophy and diastolic dysfunction in hypertensive animals. Moreover systolic LV function was also preserved in FGY-120 group. Increased intima-media thickness and interstitial fibrosis were not significantly diminished in great vessels. FGY-1153 treatment inhibited the expression of TGFβ and the phosphorylation of SMAD2 in the heart. Our results suggest that the tested novel anti-inflammatory compound has no deleterious effect on CV system, moreover it exerts moderate protective effect against the development of hypertensive cardiopathy.

## Introduction

It is well known, that the worldwide most commonly prescribed pain relievers, the NSAIDs can significantly increase cardiovascular morbidity and mortality (Patrono and Baigent, 2014). Only the low dose aspirin treatment – without significant painkiller moiety – has positive effect on survival (Patrono et al., 2005). This unfavorable phenomenon is partly caused by the increase in atherothrombotic events (Garcia Rodriguez et al., 2008; McGettigan and Henry, 2011), however, the other major cause is the increase in mortality due to heart failure (Garcia Rodriguez and Hernandez-Diaz, 2003). Therefore there is an unmet medical need to develop novel and safer anti-inflammatory compounds.

The kallikrein–kinin system is one of the multiple systems which play a role in initiation, propagation and maintenance of inflammation and pain (Moreau et al., 2005). Kinins stimulate the synthesis and release of nitric oxide, cytokines, arachidonic acid, leukotrienes as well as chemotactic factors (Lerner et al., 1989; Tiffany and Burch, 1989; Sato et al., 1996). Primary kinins (e.g., bradykinin) act predominantly on constitutively expressed (brady)kinin B2 receptors, however, secondary kinins with longer half-life (e.g., des-arginin bradykinin) bind mainly to B1 receptors (Farkas and Eles, 2011). Bradykinin B1 receptors are expressed at a very low level in healthy tissues, but undergo massive induction due to tissue injury or inflammation. Therefore the inhibition of these receptors can be effective in the relief of chronic pain and inflammation. Moreover the pharmacological inhibition of bradykinin B1 receptors theoretically seems to be safe because of the previously mentioned features (Marceau and Bachvarov, 1998).

Actually in several works beneficial cardiac effects of bradykinin B1 receptor antagonism were showed. In KO animals and using pharmacological blockade of kinin B1 receptors, both *ex vivo* and *in vivo* the size of myocardial or cerebral infarct size decreased markedly (Lagneux et al., 2002; Yin et al., 2007; Austinat et al., 2009). An other workgroup proved, that in a diabetic cardiomyopathy model the systolic and diastolic left ventricular (LV) function was better in bradykinin B1 KO animals than in wild type ones (Westermann et al., 2009).

So far there is no data in the literature regarding the effect of kinin B1 receptor antagonism in chronic elevated blood pressure. Hypertension is one of the most important risk factor of cardio- and cerebrovascular diseases and based on population-attributable risk, hypertension has the greatest impact on the development of heart failure (Lloyd-Jones, 2001). SHR is a widely accepted and used animal model to examine the development of hypertension-induced target organ damages (Trippodo and Frohlich, 1981). According to the literature, the beginning of the hypertension is at the age of 6 to 8 weeks, which, by the age of 30 weeks leads to significant target organ damages (Kokubo et al., 2005).

In this work we aimed to evaluate the effects of a new type anti-inflammatory drug, a bradykinin B1 receptor antagonist (FGY-1153) on the development of hypertensive target organ damages in spontaneously hypertensive rats (SHR).

## Materials and Methods

### Experimental Protocol

Forty-five male SHR (Charles River Laboratories, Budapest, Hungary) were used. The animals were 8 weeks old on arrival and weighed approximately 250–270 g. The study was started after an acclimatization period of 3 weeks. SHRs were randomly divided into three groups: Control group (*n* = 15), FGY120 group (*n* = 15) and FGY400 group (*n* = 15). The animals of FGY120 received test diet mixed with FGY-1153 at 120 ppm concentration (estimated to yield a dose level of approximately 6 mg/kg/day). The animals of FGY400 group received test diet mixed with FGY-1153 at 400 ppm concentration (estimated to a dose level of approximately 20 mg/kg/day). The special rat chow was purchased from Ssniff Spezialdiäten GmbH, Germany. The active ingredient (FGY1153) was developed by Richter Gedeon Plc., Hungary. The animals of the Control group received control diet (0 ppm concentration). The treatment with FGY-1153 started at the age of about 11-weeks. During the whole duration of the study (26 weeks) animals were treated orally, using standard rat chow containing FGY-1153 (in 120 or 400 ppm), or control rat diet. The rat chow was available to the animals *ad libitum*. SHR rats were observed daily, to achieve a description of normal activity, responsiveness to manipulation, weight, respiration, and general aspect (López et al., 2007).

Ten male Wistar-Kyoto age-matched rats (Harlan Laboratories S.r.l., San Pietro al Natisone, Italy) were used as a normotensive control group (WKY group). The animals of WKY group received control diet (0 ppm concentration). The animals were 32 weeks old on arrival and weighed approximately 380–420 g. After an acclimatization period of 2 weeks they were sacrificed.

### Investigations and Measurements

#### Food Consumption and Body Weight

During the last 2 weeks of the acclimatization period, all animals received Control test diet (0 ppm concentration) for adaptation. The quantity of food consumed by each cage of animals was measured and recorded daily during the adaptation period. The quantity of food consumed by each cage of animals was measured and recorded once weekly during the treatment period (except for age-matched WKY group).

Body weights were measured and recorded once weekly during the acclimatization and treatment periods and on the day of necropsy (except for age-matched WKY group).

#### Measurement of Blood Pressure

Non-invasive blood pressure measurements were performed on each animal on three occasions at Weeks 0, 13, and 26 of the treatment period. Blood pressure measurements were performed by a non-invasive tail-cuff method as described earlier (Kubota et al., 2006; Magyar et al., 2014). Blood pressure was measured by Hatteras SC1000 Blood Pressure Analysis System with rat species platform (Panlab, Harvard Apparatus; LE5001; except for age-matched WKY group).

#### Echocardiographic Examinations

Transthoracic two dimensional echocardiography (*n* = 7 from each groups) under inhalation anesthesia was performed as described earlier (Bartha et al., 2009), at the beginning of the experiment and on the day of sacrifice. Rats were lightly anaesthetized with a mixture of 1.5% isoflurane (Forane) and 98.5% oxygen. The chests of the animals were shaved, acoustic coupling gel was applied, and warming pad was used to maintain normothermia. Rats were imaged in the left lateral decubitus position. Cardiac dimensions and functions were measured from short- and long-axis views at the mid-papillary level by a VEVO 770 high-resolution ultrasound imaging system (VisualSonics, Toronto, ON, Canada) – equipped with a 25 MHz transducer. LV ejection fraction (EF), LV end-diastolic volume (LVEDV), LV end-systolic volume (LVESV), LV inside dimensions (LVIDd and LVIDs), E/E’ ratio and the thickness of septum and posterior wall (PW) were determined. EF (%) was calculated by: 100 × [(LVEDV – LVESV)/LVEDV]; relative wall thickness (RWT) was calculated by: (PW thickness + interventricular septal thickness)/LVIDd.

#### Investigation of Vascular and Cardiac Remodeling With Histology and Immunohistochemistry

Heart, carotid arteries and aortic segments excised for histological processing were fixed immediately after excision in buffered paraformaldehyde solution (4%) for 1 day. Five μm thick sections were cut. Slices were stained with Masson’s trichrome staining to detect the interstitial fibrosis as described earlier (Deres et al., 2014; Magyar et al., 2014).

The intima-media thickness was measured on cross sections of histological preparations from great arteries with Mirax Viewer software (version: 1.12.22.0).

For assessment of cardiac fibrosis, five samples were taken from all cross-sectioned cardiac preparations at constant magnification from separate, continuous territories excluding perivascular, sub-endo- and sub-pericardial areas. Different stainings were discriminated by Color deconvolution plugin for ImageJ with built-in color vectors for Masson’s trichrome. After autothresholding, the area fraction of aniline-blue staining was measured in each sample and ANOVA analysis was performed.

For evaluation of vascular fibrosis, five different areas (ROIs) from the territories of the tunica media layers of aorta and carotid arteries were selected, where measurements have been made. Different stainings were discriminated by Color deconvolution plugin for ImageJ with built-in color vectors for Masson’s trichrome. After autothresholding, the area fraction of aniline-blue staining has been measured in each predefined ROIs. On carotis data Square root transformation has been made.

All histological samples were examined by an investigator in a blinded fashion.

#### Evaluation of Cardiac and Vascular Remodeling With Electron Microscopy

Electron microscopic examinations were performed on hearts and great vessels from the treated and control SHR animals. The fixative was supplemented with 1% glutaraldehyde. After washing, samples were stained with 1% OsO_4_ in PB, dehydrated through ascending ethanol series and embedded in Durcupan ACM resin. Sections were cut, counterstained with Reynold’s lead citrate, and examined and photographed in a JEOL 1200 EX electron microscope. All histological samples were examined by an investigator in a blinded fashion.

#### Western Blot Analysis on Heart and Great Vessels

Heart samples, carotid arteries and aortic segments were homogenized in ice-cold 50 mM Tris-buffer, pH 8.0 (containing Protease and Phosphatase Inhibitor Cocktail 1:1000, Sigma Aldrich – P88340, P5726 and 50 mM sodium vanadate) and harvested in 2× concentrated SDS-polyacrylamide gel electrophoresis sample buffer. Proteins were separated on 10 or 12% SDS-polyacrylamide electrophoresis gel and transferred to nitrocellulose membranes. After blocking (2 h with 3% non-fat milk in Tris-buffered saline), membranes were probed overnight at 4°C with antibodies recognizing the following antigens: anti-N-terminal domain of beta-actin (1:10,000; Cell Signaling Technology, 3700S), extracellular signal regulated kinase (ERK 1/2; 1:1000; Cell Signaling Technology, 9170S) and phospho-specific ERK 1/2 Thr^202^/Tyr^204^ (1:1000; Cell Signaling Technology, 4370S), c-Jun N-terminal kinase (JNK; 1:1000; Cell Signaling Technology, 3708S) and phospho-specific JNK Thr^183^/Tyr^185^ (1:1000; Cell Signaling Technology, 9255S), TGF-beta (1:1000; Cell Signaling Technology, 3711S), SMAD2 (1:000; Invitrogen, 436500) and phospho-specific SMAD2 Ser^465/467^ (1:1000; Invitrogen, MA5-15122), p38 MAPK (1:000; Invitrogen, AOH1202) phospho-specific p38-MAPK Thr^180^-Gly-Tyr^182^ (1:1000; Invitrogen, MA5-15177), Akt (1:1000; Cell Signaling Technology, 9272S) and phospho-specific Akt Ser^473^ (1:1000; Cell Signaling Technology, 9018S), GSK-3-beta (1:000; Signaling Technology, 9315S) and phospho-specific GSK-3-beta Ser^9^ (1:1000; Cell Signaling Technology, 9323S). Membranes were washed six times for 5 min in Tris-buffered saline (pH 7.5) containing 0.2% Tween (TBST) before addition of goat anti-rabbit horseradish peroxidase-conjugated secondary antibody (1:3000 dilution). The antibody–antigen complexes were visualized by means of enhanced chemiluminescence. After scanning, results were quantified by NIH Image J program.

#### Blood Sampling

To assess drug plasma exposures, blood samples were collected from the rats by direct cardiac puncture on the last day of the study just before necropsy between 08.20 am and 12.20 pm. The blood samples were drawn into either heparinized tubes or Lavender Vacutainer tubes containing EDTA. The blood samples were centrifuged at 1600 *g* for 15 min at 4°C to separate plasma. Supernatants were collected and kept at -70°C.

#### Statistical Analysis

All data are expressed as mean ± SEM. For comparison of WKY and Control groups, independent sample’s *t*-test (2-tailed) was applied. Control and Treatment groups were analyzed using one-way ANOVA (SPSS for Windows 20.0). For *post hoc* comparison Dunnett’s test (2-tailed) was chosen. In cases of inhomogeneous group variances Welch correction was applied followed by Dunnett T3 *post hoc* test. On GCL cell number data Kruskal–Wallis test was conducted, due to extreme non-normal distribution of the FGY400 group. Values of *p* < 0.05 were considered statistically significant. All individual data are tabulated in the Appendix.

## Results

### Effect of FGY-1153 on Body Weight and Food Consumption

**FIGURE 1:**
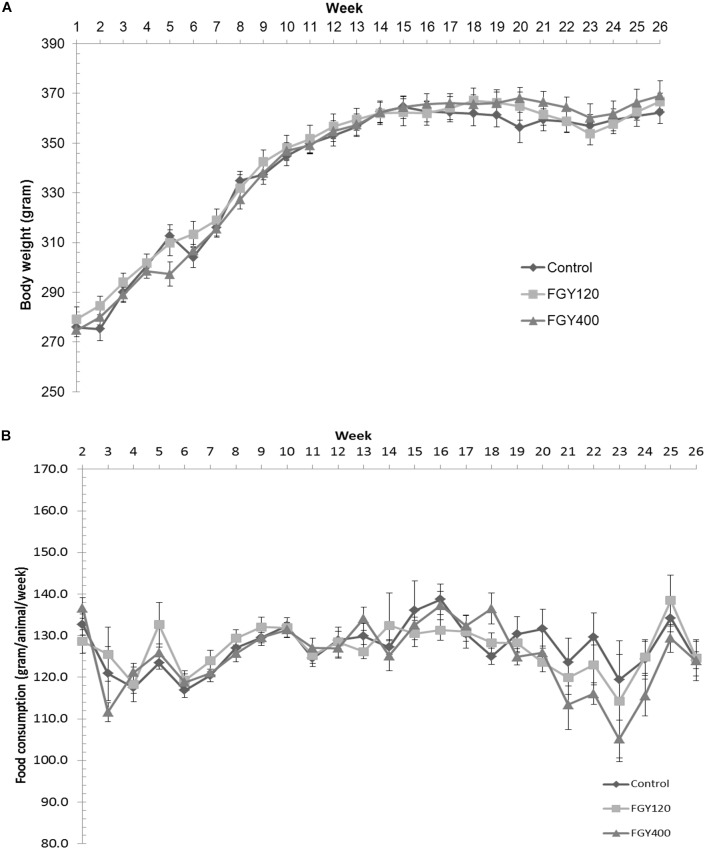
Effect of FGY-1153 on body weight and food consumption during the treatment period. **(A)** Effect of FGY-1153 on body weight during the treatment period (*n* = 14 Control, *n* = 15 FGY120, *n* = 15 FGY400). Data are presented as mean ± S.E.M. One-way ANOVA analysis conducted for each week did not reveal statistically significant differences between groups. **(B)** Effect of FGY-1153 on food consumption during the treatment period (*n* = 7 Control, *n* = 7 FGY120, *n* = 7 FGY400). Data are presented as mean ± S.E.M. Data were analyzed with one-way ANOVA. No statistically significant differences were found at any time points between the groups.

Body weights were measured and recorded once weekly during the treatment period. There were no significant differences between the three hypertensive groups neither at the beginning nor at the end of treatment period (Control: 362.4 ± 4.4 g, FGY120: 366.8 ± 3.3 g, FGY400: 369.1 ± 5.8 g) (Figure 1). The quantity of food consumed by each cage of animals was measured and recorded once weekly during the treatment period. There were no overt differences between the food consumptions of the three groups throughout the study (Figure 1 and Supplementary Tables S1–S6).

### FGY-1153 Dose Calculation and Drug Plasma Concentrations Detected

The weekly calculated dose (calculation based upon: food consumption, FGY-1153 concentration in rat chow, body weight) of test substance was 6.32 ± 0.14 (ranged from 5.51 ± 0.47 to 7.76 ± 0.14) mg/kg/day in the FGY120 group and 20.9 ± 0.59 (ranged from 16.6 ± 0.4 to 28.0 ± 0.3) mg/kg/day in the FGY400 group (Supplementary Tables S7, S8, *n* = 15).

Plasma concentrations measured from the rats of the FGY120 and FGY400 groups are presented in Supplementary Table S9.

### Effect of FGY-1153 on Blood Pressure

At the beginning of the study there was no significant difference between the systolic arterial blood pressure of the three hypertensive groups (Control: 178.73 ± 3.25 mmHg, FGY120 group: 172.47 ± 3.81 mmHg, FGY400 group: 174.53 ± 2.30 mmHg, *p* = 0.374). Systolic arterial blood pressure values did not differ significantly between the groups both at Week 13 (Control: 215.93 ± 6.11 mmHg, FGY120 group: 212.73 ± 5.68 mmHg, FGY400 group: 228.80 ± 4.48 mmHg, *p* = 0.096) and at Week 26 (Control: 256.36 ± 8.04 mmHg, FGY120 group: 256.80 ± 7.69 mmHg, FGY400 group: 275.33 ± 3.07 mmHg, *p* = 0.078; *n* = 15).

Nevertheless, a non-significant trend of higher blood pressure in the FGY400 group was apparent (Supplementary Table S10).

### Effect of FGY-1153 on Echocardiographic Parameters

Compared to the parameters measured at the beginning of the study, the septum and PW thicknesses increased in all hypertensive groups during the treatment period (*p* < 0.01). However, treatment with both low dose and high dose FGY-1153 significantly attenuated the hypertrophy of septum and PW (*p* < 0.01 and *p* < 0.05, respectively vs. Control).

**FIGURE 2:**
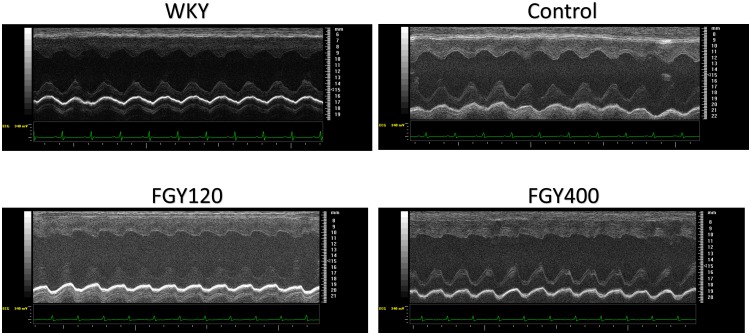
Representative M-mode pictures of the left ventricle. M-mode pictures of each group: WKY, Control, FGY120, FGY400.

LVIDs and LVIDd were also increased in all SHR groups during the study, but the elevation of these parameters were significantly attenuated in the FGY120 group (*p* < 0.05), while not in the FGY400 group. Left ventricular systolic function – expressed as EF% – showed a decreasing tendency in both the Control group and the FGY400 group by the end of the study compared to the initial parameters (Table 1). In comparison with the Control group, these changes were significantly attenuated in the FGY120 group (*p* < 0.05 vs. Control), indicating that the low dose FGY-1153 treatment prevented the hypertension induced decrease in systolic left ventricular function. The E/E’ ratio showed an increasing tendency during the study in the Control group, while this parameter was significantly decreased in both the FGY120 and FGY400 groups (*p* < 0.05 vs. Control) (Table 1 and Figure 2).

**Table 1:** Evaluation of echocardiographic parameters.

	SHR week 0	Control week 26	FGY120 week 26	FGY400 week 26	WKY age-matched
Septum (mm)	1.66 ± 0.01	2.09 ± 0.04	1.90 ± 0.04^∗∗^	1.88 ± 0.02^∗∗^	1.67 ± 0.07^∗∗^
Post. Wall (mm)	1.58 ± 0.02	1.94 ± 0.02	1.82 ± 0.01^∗^	1.81 ± 0.04^∗^	1.644 ± 0.11^∗^
LVIDd	7.28 ± 0.07	8.28 ± 0.08	7.94 ± 0.09^∗^	7.98 ± 0.11	8.00 ± 0.25
LVIDs	4.40 ± 0.07	5.38 ± 0.09	4.85 ± 0.08^∗∗^	5.12 ± 0.14	4.52 ± 0.12^∗∗^
LVEDV (ml)	280.49 ± 6.06	373.54 ± 8.11	340.79 ± 9.25^∗^	344.72 ± 10.78	349.85 ± 24.66
LVESV (ml)	88.72 ± 3.33	141.56 ± 5.89	111.69 ± 4.15^∗∗^	127.31 ± 8.66	97.07 ± 5.54^∗∗^
EF (%)	68.48 ± 0.75	62.16 ± 1.24	67.10 ± 1.33^∗^	63.36 ± 1.37	71.67 ± 0.87^∗∗^
E/E’	35.16 ± 1.54	42.17 ± 5.26	30.05 ± 0.86^∗^	26.50 ± 2.77^∗^	30.00 ± 2.26
RWT	0.447 ± 0.004	0.485 ± 0.014	0.469 ± 0.007	0.464 ± 0.011	0.413 ± 0.01^∗∗^

*Data of all animals are presented in the first column (SHR Week 0, N = 21) at the beginning of the study and data from the three groups (Control, FGY120, FGY400 Week 26; n = 7 in each groups) are indicated at the end of the treatment period. Last column represents the data of age-matched normotensive animals (WKY, n = 7). Values are expressed as mean ± S.E.M. Comparisons between WKY and Control groups were made by independent samples t-test. Data of Control and Treatment groups were analyzed with one-way ANOVA followed by Dunnett’s post hoc test (^∗^p < 0.05, ^∗∗^p < 0.01 vs. Control).*

### Effect of FGY-1153 on the Interstitial Fibrosis of Heart and Great Vessels

**FIGURE 3:**
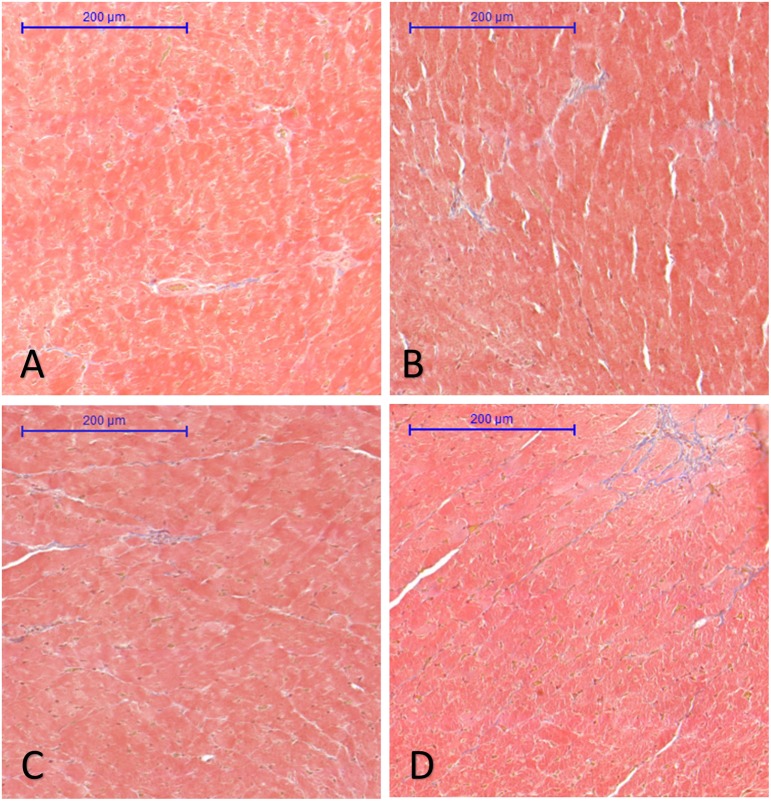
Effect of FGY-1153 treatment on the interstitial cardiac fibrosis. Statistical analysis of Masson’s trichrome stained sections revealed no significant difference between the Control **(B)**, FGY120 **(C)**, and FGY400 **(D)** groups. WKY group **(A)**, however, showed significantly lower collagen content relative to Control **(B)** group (WKY: 0.390 ± 0.021; Control: 0.657 ± 0.069; FGY120: 0.636 ± 0.088; FGY400: 0.582 ± 0.041; *n* = 4).

Analysis of interstitial fibrosis in SHR heart samples revealed no statistically significant difference between Control and Treatment groups (FGY120 and 400). The collagen content in WKY hearts, however, was significantly lower (*p* < 0.05) compared to the Control group (Mean area fractions ± SEM: WKY: 0.390 ± 0.021; Control: 0.657 ± 0.069; FGY120: 0.636 ± 0.088; FGY400: 0.582 ± 0.041; *n* = 4) (Figure 3). In carotid arteries and aortas a statistically non-significant increase of vascular collagen could be observed in Control group compared to WKY. No significant differences could be found between Control, FGY120 and FGY400 groups (Mean area fractions ± SEM: Aorta: WKY: 1.084 ± 0.112 (*p* = 0.536 vs. Control); Control: 1.378 ± 0.414; FGY120: 1.239 ± 0.526; FGY400: 1.458 ± 0.324, (*p* = 0.936); *n* = 4). In carotid arteries a statistically non-significant decrease of vascular collagen could be observed in treated groups: WKY: 4.860 ± 0.532 (*p* = 0.229 vs. Control); Control: 5.994 ± 0.660; FGY120: 5.745 ± 1.465; FGY400: 5.158 ± 1.097; (*p* = 0.866), *n* = 4 data not shown).

### Effect of FGY-1153 on the Intima-Media Thickness of Great Vessels

**FIGURE 4:**
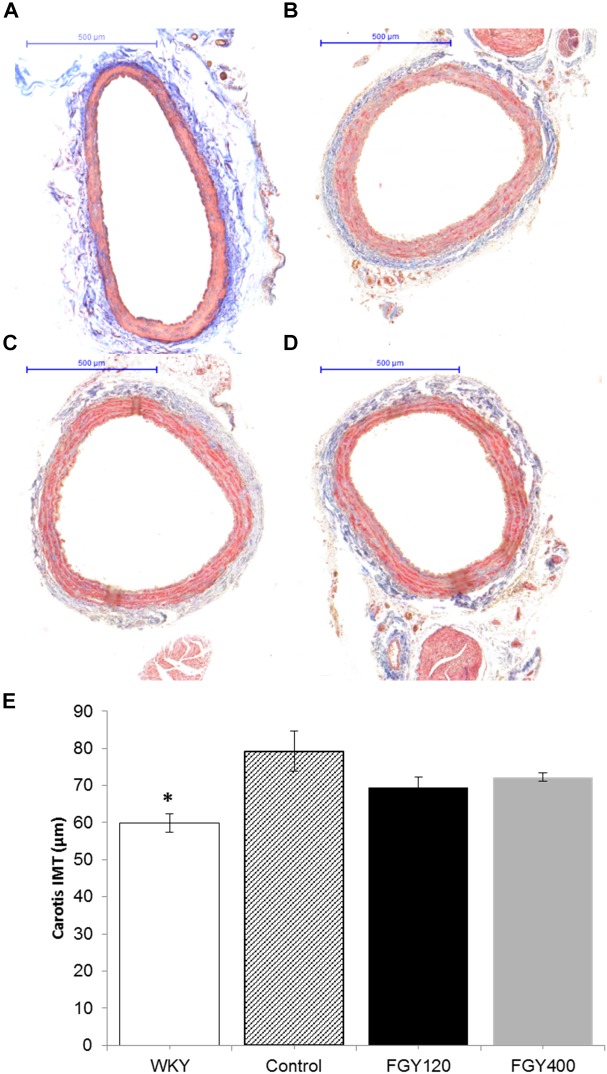
Masson’s trichrome staining of carotid vessels. IMT was the lowest in the WKY group **(A)**. In comparison with the Control group **(B)**, the intima-media thickness of carotid vessels was slightly decreased in both the FGY120 **(C)** and FGY400 groups **(D)**, (*n* = 4). The alterations were not significant. Effect of FGY-1153 treatment on intima-media thickness of carotid vessels **(E)**. IMT, intima-media thickness (^∗^*p* < 0.05 vs. Control group (*n* = 3).

The IMT of carotid arteries was the lowest in the normotensive WKY group, (Figure 4A,B,E) (*p* < 0.05 vs. Control *n* = 3). Chronic hypertension caused a marked increase of IMT in the Control group. In comparison with the Control group (Figure 3B), the intima-media thickness of carotid vessels was slightly decreased in both the FGY120 and FGY400 groups (Figure 4C,D). However, the alterations were not statistically significant (*p* = 0.149, *n* = 4).

### Effect of FGY-1153 on the Ultrastructural Changes in the Myocardium and Great Vessels

#### Electron Microscopic Studies of the Myocardium

**FIGURE 5:**
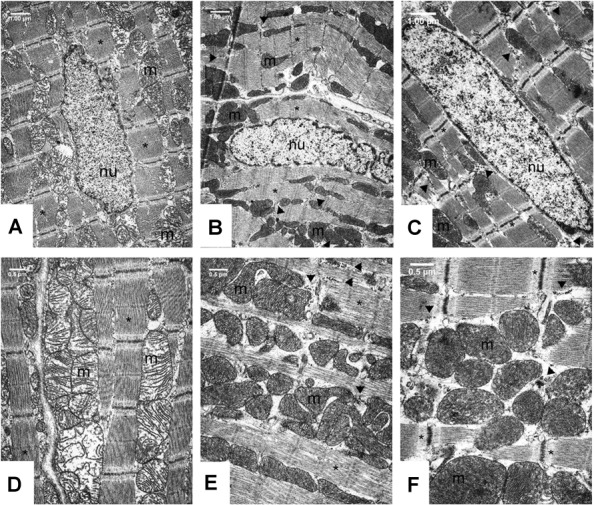
Ultrastructural analysis of the cardiomyocytes in Control **(A,D)**, FGY120 **(B,E)**, and FGY400 **(C,F)** group. Sarcoplasm (sp), Muscle fibers (^∗^), Mitochondria (m), Nuclei (nu), Glycogen (arrow heads), (*n* = 5 from each group, 3–5 block from each animal).

Transmission electron microscopic analysis of capillary endothelium in the myocardium (Supplementary Figures S1, S2) showed normal structure in all groups. The morphology of Eberth’ lines (not shown) and sarcomere units was also normal in both the Control and FGY-1153 treated groups (Supplementary Figures S1D–F). The nuclei of cardiomyocytes in the Control rats predominantly display euchromatin with minor membrane-associated marginal heterochromatin (Figure 5A). In contrast, in the other two groups cell nuclei were pale and displayed higher amount of membrane-associated marginal chromatin suggesting differences in the activity of transcription (Figure 5B,C). In the cytoplasm glycogen (Figure 5 and Supplementary Figures S1, S2 arrow heads) and lipid droplets (not shown) could be seen in all groups, however, the amount of glycogen was slightly increased and the number of lipid droplets was slightly decreased in the FGY120 group compared to the Control and FGY400 groups.

The mitochondria of SHR rats (Figure 5) differ from the normal mitochondria of healthy rats (Supplementary Figure S2). In the Control group (Figure 5D) extensive disruption of mitochondrial cristae and enlarged intracristal spaces could be observed. The mitochondrial population was characterized by morphological heterogeneity, their structure was polymorph, their shape was often elongated, and the mitochondrial matrix was very light.

The mitochondrial ultrastructure in the FGY120 group was similar to that of the healthy rats (Figure 5E and Supplementary Figure S2). The structure of the mitochondrial cristae was almost normal, dense matrix was seen and the mitochondria were less elongated. The ratio of mitochondria to myofibrils was higher, the elevated number of mitochondria was accompanied by increased amount of intracellular glycogen in comparison to the Control group. In the samples of FGY400 group (Figure 5F) the mitochondrial matrix was also dense, however, more morphological abnormalities, e.g., disorganized mitochondrial cristae could be revealed in comparison to the FGY120 group (*n* = 5 from each group, 3–5 block from each animal).

### Electron Microscopic Studies of the Walls of Aortas and Carotid Arteries

The ultrastructure of the wall of normotensive aorta (not shown) and carotid arteries is characterized by endothelial cells lying almost directly on the internal elastic lamina (Supplementary Figure S3). The surface of the endothelial monolayer is smooth, the cells have very thin cytoplasm and an elongated nucleus which is oriented parallel to the internal elastic lamella. The subendothelial space is narrow, the internal elastic lamina is rarely interrupted. In the tunica intima the cellular elements (fibroblasts, smooth muscle cells) are arranged between the layers of internal elastic lamina (Supplementary Figure S3).

**FIGURE 6:**
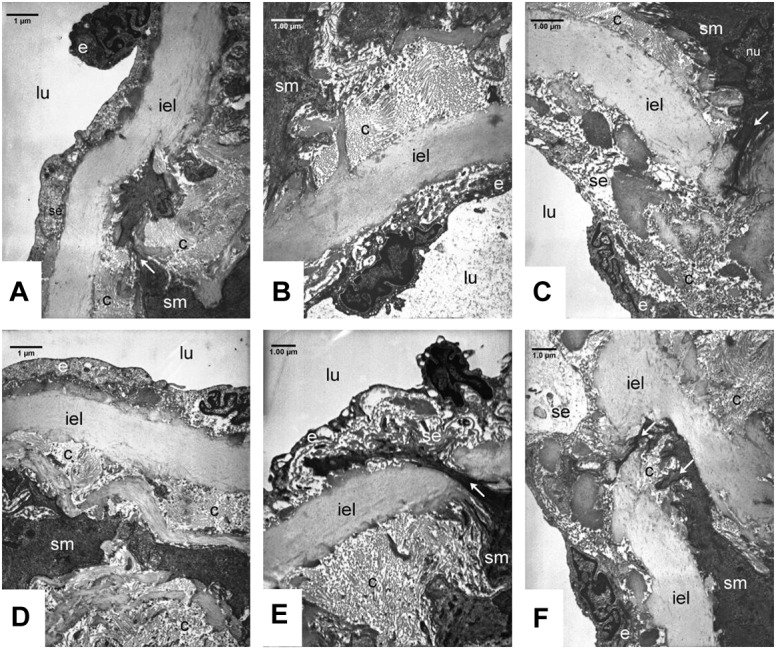
Ultrastructural analysis of carotid walls in the Control **(A, D)**, FGY120 **(B, E)**, and FGY400 **(C, F)** groups. Lumen (lu), Endothelium (e), Subendothelium (se), Internal elastic lamina (iel), Smooth muscle cells (sm), Collagen fibers (c), Interruption of internal elastic lamella by the smooth muscle cells (arrows), (*n* = 5 from each group, 3–5 block from each animal).

In the carotid arteries (Figure 6) and also in the aorta (data not shown) of the SHR groups the wall structure was pathologic. The nuclei of smooth muscle cells in the intimal layer were lobular and showed signs of activation. The activated smooth muscle cells are involved in the synthesis of the components of fibers, which was confirmed by the presence of collagen and elastic fibers around them (Figure 6). Expanding smooth muscle cells sporadically broke through the internal elastic lamina (Figure 6C,E,F), resulting in the appearance of collagen and cellular elements in the subendothelial space. Accumulation of connective tissue and cellular elements in the subendothelial space can exert tension to the endothelial monolayer, so the endothelial cells became distorted, their cytoplasm was lateralized and in some places became thinner. Presumably the endothelial permeability increased due to cell distortion, which contributed to subendothelial space expansion and further endothelial erosion.

Additional sign of tension-distorted endothelial cells is the altered orientation of their nuclei, which became rounded and protruded into the lumen, so the surface of the endothelial monolayer became rough (Figure 6A,B,E).

Rearrangement of tunica media layers could be seen, as a consequence of hypertension-induced smooth muscle cell activation and collagen synthesis. In the walls of carotid arteries more intensive collagen synthesis was seen compared to aortic walls. We could not see any significant differences between the control group and the treated groups (FGY120 and FGY 400) (Figure 6) (*n* = 5 from each group, 3–5 block from each animal).

### Effect of FGY-1153 on the TGFβ/SMAD2 Signaling Pathway in Heart and Great Vessels

#### Western Blot Analysis of Heart Samples

**FIGURE 7:**
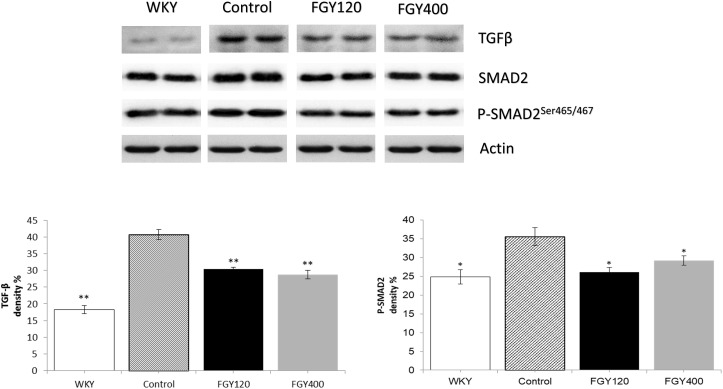
The effect of FGY-1153 on the TGFβ/SMAD2 signaling pathway in heart samples. Data are presented as mean ± S.E.M. Data were analyzed by independent samples *t*-test between WKY and Control groups. Comparisons of Control and Treatment groups were made by one-way ANOVA followed by Dunnett’s *post hoc* test. ^∗^*p* < 0.05, ^∗∗^*p* < 0.01 vs. Control, *n* = 4.

Western blot analysis showed that both TGFβ and SMAD2 phosphorylation levels were significantly lower in WKY animals compared to the Control group. FGY-1153 treatment inhibited the cardiac expression of TGFβ and the phosphorylation of the SMAD2 protein in the FGY120 group, however, the high dose treatment had no significant effect on the phosphorylation of SMAD2 in the FGY400 group. Actin is shown as loading control. Representative immunoblots from four experiments and densitometric evaluation are demonstrated (Figure 7).

#### Western Blot Analysis of Carotid Samples

**FIGURE 8:**
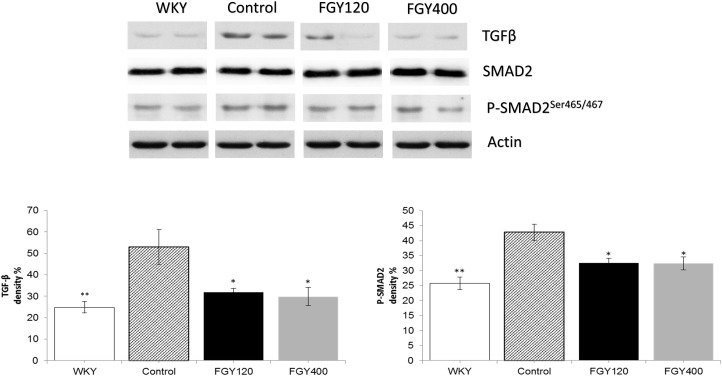
The effect of FGY-1153 on the TGFβ/SMAD2 signaling pathway in the carotid arteries. Data are presented as mean ± S.E.M. Data were analyzed by independent samples *t*-test between WKY and Control groups. Comparisons of Control and Treatment groups were made by one-way ANOVA followed by Dunnett’s *post hoc* test. ^∗^*p* < 0.05, ^∗∗^*p* < 0.01 vs. Control *n* = 4.

Western blot analysis showed that both TGFβ expression and SMAD2 phosphorylation levels were significantly higher in Control group relative to WKY. Both low and high dose FGY-1153 treatment significantly inhibited the expression of TGFβ. The phosphorylation of the SMAD2 protein was significantly decreased in both the FGY120 and in FGY400 groups in carotid tissues. Actin is shown as loading control. Representative immunoblots from four experiments and densitometric evaluation are demonstrated (Figure 8).

### Effect of FGY-1153 on the Phosphorylation of MAPK Signaling Cascade in Heart and Great Vessels

#### Western Blot Analysis of Heart Samples

**FIGURE 9:**
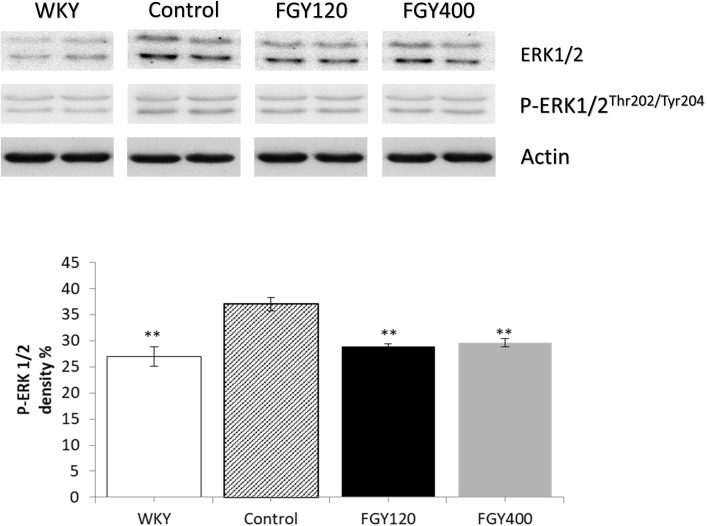
The effect of FGY-1153 on the ERK 1/2 signaling in heart of SHR rats. Data are presented as mean ± S.E.M. Data were analyzed by independent samples *t*-test between WKY and Control groups. Comparisons of Control and Treatment groups were made by one-way ANOVA followed by Dunnett’s *post hoc* test. ^∗∗^*p* < 0.01, *n* = 4.

Western blot analysis showed significantly lower ERK1/2 phosphorylation level in WKY, relative to the Control group (Figure 9). FGY-1153 treatment significantly inhibited ERK1/2 phosphorylation in both FGY120 and in FGY400 hearts. However, it had no statistically significant effect on the phosphorylation of p38-MAPK and JNK proteins (data not shown).

#### Western Blot Analysis of Carotid Samples

**FIGURE 10:**
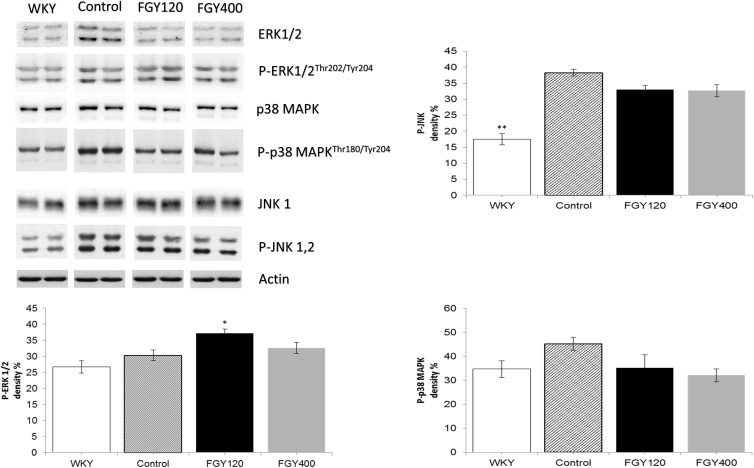
The effect of FGY-1153 on the MAPK signaling factors in carotid vessels of SHR rats. Data are presented as mean ± S.E.M. Data were analyzed by independent samples *t*-test between WKY and Control groups. Comparisons of Control and Treatment groups were made by one-way ANOVA followed by Dunnett’s *post hoc* test. ^∗^*p* < 0.05, ^∗∗^*p* < 0.01, *n* = 4.

Phosphorylation level of JNK was significantly higher in the Control relative to the WKY group. Western blot analysis showed that low dose FGY-1153 treatment had no significant effect on phosphorylation of p38-MAPK and JNK proteins, but elevated the phosphorylation level of ERK1/2 protein. High dose treatment had no significant effect on the phosphorylation of MAPKs. Actin is shown as loading control. Representative immunoblots from four experiments and densitometric evaluation are demonstrated (Figure 10).

### Effect of FGY-1153 on the Phosphorylation of Akt/GSK-3β Signaling Cascade in Heart and Great Vessels

#### Western Blot Analysis of Heart Samples

Western blot analysis showed GSK-3β phosphorylation to be significantly lower in WKY relative to Control group. High dose of FGY-1153 treatment significantly elevated phosphorylation of Akt protein, and both low dose and high dose treatment significantly attenuated the GSK-3β phosphorylation (data not shown).

#### Western Blot Analysis of Carotid Samples

**FIGURE 11:**
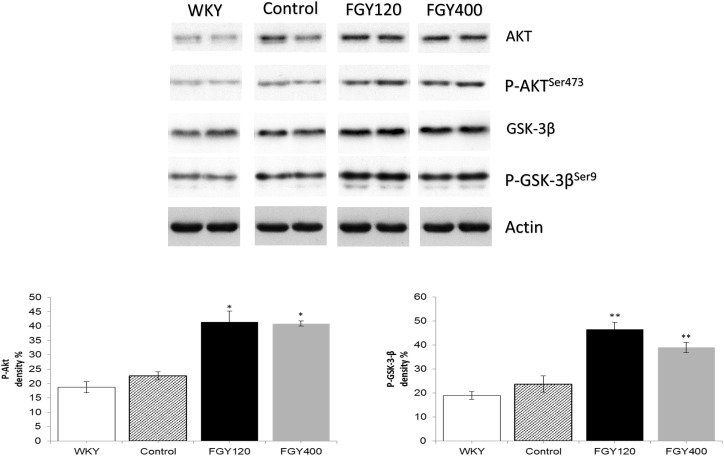
The effect of FGY-1153 on the Akt/GSK-3β signaling cascade in carotis wall. Data are presented as mean ± S.E.M. Data were analyzed by independent samples *t*-test between WKY and Control groups. Comparisons of Control and Treatment groups on GSK-3β data were made by one-way ANOVA followed by Dunnett’s *post hoc* test. On Akt data one-way ANOVA with Welch correction were conducted followed by Dunnett T3 *post hoc* test. ^∗^*p* < 0.05, ^∗∗^*p* < 0.01 vs. Control, *n* = 4.

Western blot analysis of Akt protein and GSK-3β phosphorylation showed no difference between the WKY and control SHR groups. FGY-1153 treatment significantly promoted the phosphorylation of Akt protein and GSK-3β in the carotid tissues of both FGY120 and FGY400 groups. However, the high dose treatment had milder effect on the GSK-3β phosphorylation in the FGY400 group. Actin is shown as loading control. Representative immunoblots from four experiments and densitometric evaluation are demonstrated (Figure 11).

## Discussion

The major finding of this study is that pharmacological inhibition of bradykinin B1 receptors do not cause elevation of blood pressure and do not cause worsening of hypertensive target organ damages in a chronic hypertensive animal model. Moreover, we have found that the administration of FGY-1153, an experimental bradykinin B1 receptor antagonist compound causes slight protective effect against cardiovascular remodeling. We used SHR which provide an animal model of high blood pressure that is similar to essential hypertension in humans (Ito et al., 2007).

Hypertension is a growing public health problem mainly in middle-aged and in elderly people. In hypertensive patients systolic blood pressure (SBP) and pulse pressure are the dominant prognostic markers. Plenty of studies showed that increasing levels of SBP were associated with a higher risk of cerebro- and cardiovascular events. Unfortunately, the rate of hypertension control only minimally improved over the last decades. One important factor in the background of the inadequate hypertension control is the drug interactions between antihypertensive agents and several non-cardiovascular drugs, e.g., NSAIDs (Pavlicević et al., 2008; Deedwania, 2011).

Moreover, it is also well-known, that the worldwide commonly prescribed pain relievers, the NSAIDs can significantly increase cardiovascular morbidity and mortality (Patrono and Baigent, 2014). This unfavorable phenomenon is partly caused by the increase in atherothrombotic events (Garcia Rodriguez et al., 2008; McGettigan and Henry, 2011), but the other major cause is the increase in mortality due to heart failure (Garcia Rodriguez and Hernandez-Diaz, 2003).

Because of the above mentioned causes there is an unmet medical need to develop novel and safer anti-inflammatory compounds.

Kinins are biologically active peptides that exert a broad spectrum of physiological effects, including vasodilation, smooth muscle contraction, inflammation, and pain induction (Tschöpe et al., 2000). The biological effects of kinins are mediated through the stimulation of bradykinin B1 and B2 receptors. The B2 receptor is constitutively expressed and is activated by intact kinins, bradykinin, and kallidin. This receptor is believed to play an important role in mediating the beneficial effects of ACE-inhibitors, but it is also involved in the acute phases of inflammation (Tschöpe et al., 2000; Leeb-Lundberg et al., 2005; Golias et al., 2007). However, the B1 receptor is activated by the carboxypeptidase metabolites of kinins, des-Arg9-BK and des- Arg10-kallidin. The B1 receptor is normally weakly expressed, but it is upregulated in the presence of cytokines, endotoxins or during tissue injury (Tschöpe et al., 2000; Leeb-Lundberg et al., 2005; Golias et al., 2007). The B1 receptor mediates chronic inflammation and pain (Leeb-Lundberg et al., 2005; Golias et al., 2007); thus, bradykinin B1 receptor antagonism can be a potential novel approach for treating these conditions without having deleterious cardiovascular effects.

On this basis, we examined the effect of FGY-1153, a bradykinin B1 receptor antagonist (administered in two concentrations) on the cardiovascular changes provoked by chronic hypertension.

FGY-1153 treatment did not have any effects on food consumption and on body weight. The mean daily doses of FGY-1153 in the 120 ppm and 400 ppm groups were approximately 6 mg/kg and 20 mg/kg, respectively.

Chronic onset of elevated blood pressure in hypertension leads to the development of hypertensive cardiopathy (Montezano et al., 2015). This pathology of heart is characterized morphologically by marked thickening of left ventricular walls, also known as left ventricular hypertrophy. Functionally these hearts show diastolic dysfunction and if the hypertension is not treated properly, finally the contractile function will also be impaired (Nadruz, 2015). In our work there was no significant difference between the blood pressures in the three SHR groups, moreover, the high dose treatment (20 mg/kg/day) caused a non-significant increase in blood pressure. In spite of the unchanged or even slightly higher blood pressures, FGY-1153 had a mild but significant protective effect against the development of hypertension-induced left ventricular hypertrophy. The thickness of LV walls (septum and PW), as well as the RWT were lower in treated hypertensive groups. One of the best measures of diastolic function is the E/E’ ratio (Sharifov et al., 2016). The higher the ratio, the worse the diastolic function. The highest ratios could be seen in the Control group and they were decreased in animals treated by the bradykinin B1 receptor antagonist compound. The EF is the best indicator of LV systolic function (Cameli et al., 2016). Chronic hypertension caused a significant decreased EF in the Control group compared to normotensive animals. Low dose FGY1153 treatment prevented the hypertension-induced decrease of EF, but there was only an improving tendency in EF in the high-dose group (FGY400).

In spite of the above mentioned positive effects, in our experimental model, the bradykinin B1 receptor antagonist treatment had only marginal, non-significant effect on hypertension-induced vascular wall thickening in great vessels characterized by IMT.

The increased thickness of left ventricle and vascular walls in hypertensive patients is determined by the hypertrophy of cardiomyocytes/vascular smooth muscle cells and by the amount of interstitial collagen accumulation (Montezano et al., 2015). The myocardial and vascular collagen content was examined by transmission electron microscopy and measured by Masson’s trichrome staining. FGY-1153 treatment did not decrease significantly the extent of hypertension-induced myocardial and vascular fibrosis (it only caused an improving tendency) (Limas et al., 1980; Okruhlicová et al., 2008; Sandow et al., 2009).

In the background of the cardioprotective effect of pharmacological bradykinin B1 blockade the preservation of mitochondrial structure can be the main cause. In untreated hypertensive animals (Control group) mitochondrial structure was disorganized, the cristae were disrupted, the intracristal spaces were enlarged and the sizes of mitochondria were heterogeneous. FGY-1153 treatment – predominantly the lower dose – maintained the normal structure of mitochondria in spite of chronic elevated blood pressure. This is a very important effect of FGY-1153, because mitochondria are one of the most important sources of reactive oxygen species formation and oxidative stress plays a central role in the pathogenesis of hypertensive organ damages (Puddu et al., 2008; Cal et al., 2011). It is well known, that the prosurvival signaling pathway (PI3K/Akt-1/GSK-3β) exerts its protective effect via maintaining the mitochondrial structure and function (Yang et al., 2015). Measuring the activity of these factors, we could observe that FGY-1153 caused a marked phosphorylation of Akt-1 and GSK-3β signaling factors.

Damaged and disorganized mitochondria via oxidative stress can alter the activity of several signal transduction pathways that have an important role in the pathogenesis of vascular and cardiac remodeling.

Oxidative stress provoked activation of signal transduction pathways can promote remodeling. One of these signaling factors are the MAP kinases (MAPKs) (Tanaka et al., 2001). In the recent work hypertension caused an increased phosphorylation state of the various MAPKs (p38-MAPK, ERK, JNK). However, FGY-1153 treatment did not cause consistent and significant changes in the activation state of these signaling molecules. ERK1/2 activity decreased in heart samples and JNK activity was also decreased in carotid arterial samples compared to untreated ones. However, in other samples there were no differences between treated and untreated animals.

In the mediation of vascular remodeling, the TGF-β/Smad pathway also plays an important role (Ruiz-Ortega et al., 2007; Deres, 2014). In the recent work we proved that FGY-1153 caused a significant inhibition in hypertension-induced activation of TGF and Smad-2 pathways in both myocardium and vascular walls. Therefore in the background of the beneficial effects of FGY-1153 treatment (predominantly in lower dose) the modulation of this pathway can also be an important factor besides its mitochondrial protective effect (Ruiz-Ortega et al., 2007; Perrotta et al., 2011).

## Conclusion

In conclusion, the bradykinin B1 receptor antagonist compound FGY-1153 did not have any deleterious effects in SHR rats administering in low dose nor in high dose. Moreover we could observe some protective effects against hypertensive cardiovascular remodeling despite the fact that FGY-1153 did not have any antihypertensive effect. Inhibition of the TGF-β/Smad-2 and activation of Akt-1/GSK-3β signaling may be the main underlying mechanisms in the background of its cardiovascular protective effect.

## Disclosure

CC and SF was employed by company Gedeon Richter Plc., Budapest, Hungary. All other authors declare no competing interests.

## Ethics Statement

The investigation conforms to the Guide for the Care and Use of Laboratory Animals published by the U.S. National Institutes of Health and was approved by the Animal Research Review Committee of the University of Pécs, Medical School (BA02/2000-2/2010).

## Author Contributions

LD, RH, SF, CC, and KT designed the study. LD, KE, NB, OH, and RH performed the experiments. SF and CC provided compounds and rats. LD, RH, TH, KE, and OH processed and analyzed the data. LD, RH, CC, KT, and SF wrote the manuscript. All the authors reviewed and finally approved the manuscript.

## Conflict of Interest Statement

The authors declare that the research was conducted in the absence of any commercial or financial relationships that could be construed as a potential conflict of interest.
