# Supplementary material for: The Effects of Bradykinin B1 Receptor Antagonism on the Myocardial and Vascular Consequences of Hypertension in SHR Rats
Source: Front Physiol. 2019 May 21;10:624. doi: 10.3389/fphys.2019.00624 (PMC6537226; doi:10.3389/fphys.2019.00624)
Supplement: Supplementary file 1 [file Table_1.docx]

**S1 Table.** Body weights (gram) in Control group during the treatment period.

**S2 Table.** Body weights (gram) in FGY120 group during the treatment period.

**S3 Table.** Body weights (gram) in FGY400 group during the treatment period.

**S4 Table.** Food consumption in Control group during the treatment period.

**S5 Table.** Food consumption in FGY120 group during the treatment period.

**S6 Table.** Food consumption in FGY400 group during the treatment period.

**S7 Table.** Calculated dose of test substance (mg/kg/day) in FGY120 group.

**S8 Table.** Calculated dose of test substance (mg/kg/day) in FGY400 group.

**S9 Table.** Plasma concentrations measured from individual animals of FGY-1153 treated groups at the end of the study.

| FGY120 | | FGY400 | |
| --- | --- | --- | --- |
| ID | Plasma  conc.(ng/ml) | ID | Plasma  conc.(ng/ml) |
| 8a | 28 | 15a | 359 |
| 8b | 118 | 15b | 244 |
| 9a | 90 | 16a | 239 |
| 9b | 105 | 16b | 264 |
| 10a | 84 | 17a | 267 |
| 11a | 92 | 17b | 467 |
| 11b | 50 | 18a | 585 |
| 14a | 88 | - | - |
| Mean | 81.9 | Mean | 346.4 |
| S.D. | 29.2 | S.D. | 133.4 |

**S10 Table.** Effect of FGY-1153 on blood pressure.
