# Supplementary material for: The Effects of Bradykinin B1 Receptor Antagonism on the Myocardial and Vascular Consequences of Hypertension in SHR Rats
Source: Front Physiol. 2019 May 21;10:624. doi: 10.3389/fphys.2019.00624 (PMC6537226; doi:10.3389/fphys.2019.00624)
Supplement: Supplementary file 2 [file Image_1.pdf]

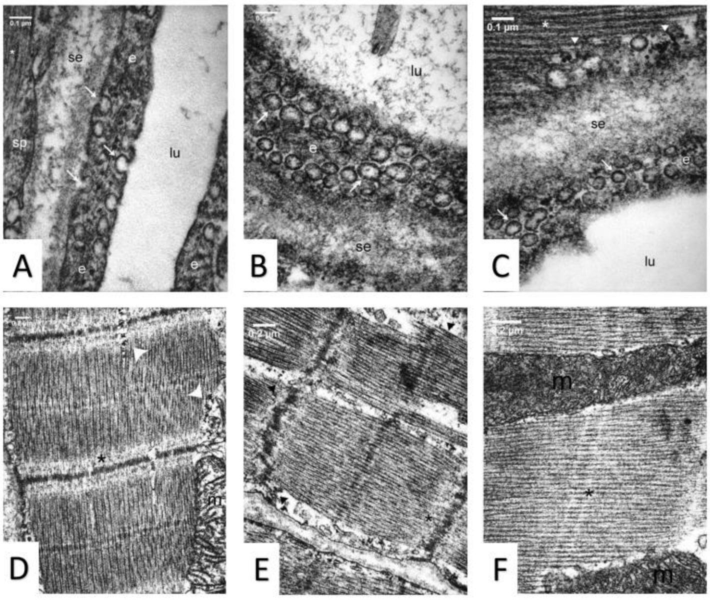

S1 Fig. Ultrastructural analysis of the cardiomyocytes in Control (A, D), FGY120 (B, E) and FGY400 (C, F) group. Endothelium (e), Lumen of endothelium (lu), Subendothelium (se), Vesicles (arrows), Sarcoplasm (sp), Muscle fibers (*), Mitochondria (m), Glycogen (arrow heads), (n=5 from each group, 3-5 block from each animal).

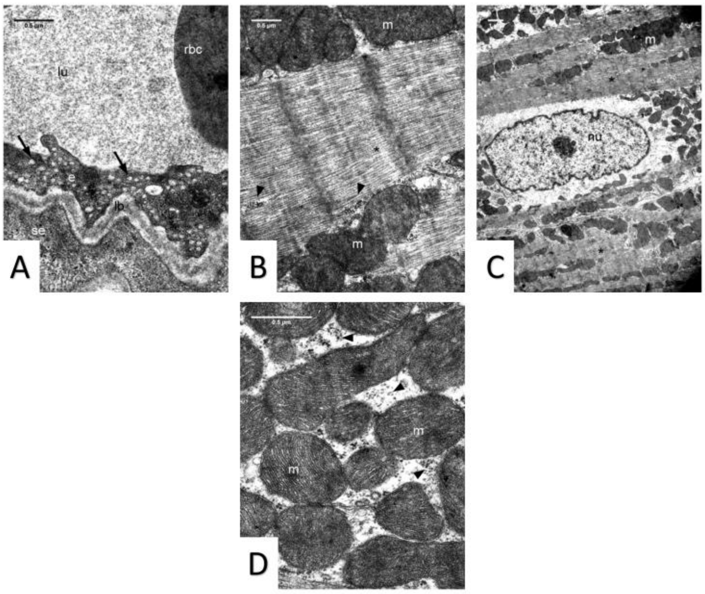

S2 Fig. Ultrastructural analysis of the cardiomyocytes in WKY group. Endothelium (e), Lumen of endothelium (lu), Subendothelium (se), Vesicles (arrows), Sarcoplasm (sp), Muscle fibers (*), Mitochondria (m), Nuclei (nu), Glycogen (arrow heads), (n=5 from each group, 3-5 block from each animal).

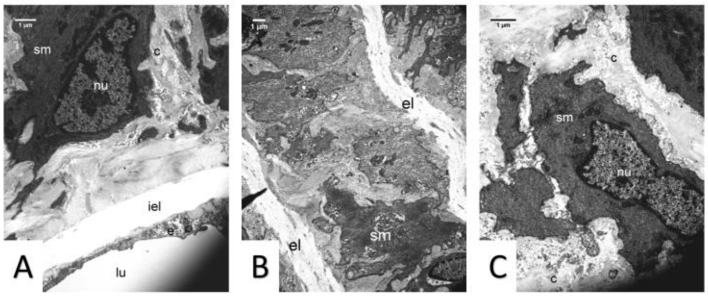

S3 Fig. Ultrastructural analysis of the aortic wall in WKY group. Lumen (lu), Endothelium (e), Subendothelium (se), Internal elastic lamina (iel), Smooth muscle cells (sm), Collagen fibers (c), Interruption of internal elastic lamella by the smooth muscle cells (arrows), (n=5 from each group, 3-5 block from each animal).
